# Wood Sawdust/Natural Rubber Ecocomposites Cross-Linked by Electron Beam Irradiation

**DOI:** 10.3390/ma9070503

**Published:** 2016-06-23

**Authors:** Elena Manaila, Maria Daniela Stelescu, Gabriela Craciun, Daniel Ighigeanu

**Affiliations:** 1 National Institute for Laser, Plasma and Radiation Physics, Electron Accelerators Laboratory, 409 Atomistilor St., Magurele 077125, Romania; elena.manaila@inflpr.ro (E.M.); daniel.ighigeanu@inflpr.ro (D.I.); 2 National R&D Institute for Textile and Leather—Leather and Footwear Research Institute, 93 Ion Minulescu St., Bucharest 031215, Romania; dmstelescu@yahoo.com

**Keywords:** natural rubber, wood sawdust, electron beam irradiation, physical and mechanical properties, cross-link density, water uptake

## Abstract

The obtaining and characterization of some polymeric eco-composites based on wood sawdust and natural rubber is presented. The natural rubber was cross-linked using the electron beam irradiation. The irradiation doses were of 75, 150, 300 and 600 kGy and the concentrations of wood sawdust were of 10 and 20 phr, respectively. As a result of wood sawdust adding, the physical and mechanical properties such as hardness, modulus at 100% elongation and tensile strength, showed significant improvements. The presence of wood sawdust fibers has a reinforcing effect on natural rubber, similar or better than of mineral fillers. An increase in the irradiation dose leads to the increasing of cross-link density, which is reflected in the improvement of hardness, modulus at 100% elongation and tensile strength of blends. The cross-linking rates, appreciated using the Flory-Rehner equation, have increased with the amount of wood sawdust in blends and with the irradiation dose. Even if the gel fraction values have varied irregularly with the amount of wood sawdust and irradiation dose it was over 90% for all blends, except for the samples without wood sawdust irradiated with 75 kGy. The water uptake increased with increasing of fiber content and decreased with the irradiation dose.

## 1. Introduction

The remarkable properties of natural rubber (NR) make it preferable for many engineering applications and maintain it as heavily investigated. It has a long fatigue life and high strength even without reinforcing fillers. For other purposes than for thin sections it can be used at approximately 100 °C, and sometimes above. In addition, it can maintain its flexibility down to −60 °C, has a good creep and stress relaxation resistance and the cost is low [1]. The most important stage in the technology of rubber obtaining is the vulcanization/cross-linking. During this process, the rubber molecules having chain configuration are linked by chemical bridges/bonds and the rubber mass turns from its plastic mass into an elastic one. For rubbers with general purposes, this is done by the addition of sulfur in the presence of organic accelerators [2,3], but it is a very complicated process [4,5]. The resulting cross-links may be mono-, di-, tri- or higher poly-sulfides, in proportions determined, among others, by the vulcanization system, the cure time or temperature. These compounds and their reaction products are allergy-causing and cytotoxic [6]. The sulfur vulcanization process requires the presence in the polymer of some unsaturated carbon–carbon links and leads to a three-dimensional rubber network in which the polymer chains are linked to each other by sulfur bridges. As a result, the sulfur-cured materials even if they have good tensile and tear strengths, good dynamic properties, have poor high temperature properties like aging, for instance [7,8]. An alternative curing system has been developed using peroxides because they are preferred when performances like extra scorch safety, shelf life, bin stability, low permanent set and high-temperature are desired. All the above curing systems involve chemicals by which the purity of the processed products is not maintained. In order to develop and use alternative and sustainable technologies, the use of electron beam (EB) irradiation for cross-linking and grafting of elastomers has become more and more attractive. The process is very clean, the energy consumption is small, permits high processing speeds than in other processes of curing and operates at ambient temperature. Starting in the 1950s, the physical and chemical changes in polymers obtained by radiation techniques, and also the involved mechanisms of cross-linking and chain scission, have been investigated. Because of their advantages, like inducing chemical reactions without catalyst at any temperature in the solid, liquid and gas phase or energy saving, the ionizing radiations are very attractive for industry [9,10]. Particularly, electron beam irradiation has shown to have many benefits compared with the conventional curing systems such as: EB cross-linking occurs at room temperature so does not appear the polymer degradation due to high temperatures involved in classical treatments, no oxidative degeneration in polymers, direct cross-linking by C–C linkage, high degree of cross-linking, extremely strong bonds, short curing cycles, high productivity, suitable for thin products and no material waste [9,10]. Currently, the reinforcement of NR used in different industrial applications is made using active fillers such as silica or carbon black. However, they are well known as having undesired effects on human health, such as as silicosis, cancer (Group 1 and 2B respectively, according to the International Agency for Research on Cancer), autoimmune diseases, tuberculosis, kidney diseases, etc. [11,12]. From all these reasons, were made efforts to replace them with other types of fillers. Very good and eco-friendly reinforcement substitutes are natural fibers, a class of renewable materials which experiencing nowadays a great revival [13]. From considerations of energy saving, favorable processing properties, occupational health benefits, cost saving, dimensional stability and not least biodegradability, the wood sawdust was considered as being a good active fillers substitute [14,15,16]. To obtaining composites, many experiments were made based on different polymeric materials and natural fibers including wood sawdust, using classical methods of cure consisting of repeated heating cycles in hot presses. A connection was established between the filler particle dimensions and mechanical properties of the composites. Thus, they present completely different tensile properties as a function of filler particle dimensions and loads. Moreover, there is a minimum critical fiber length below which the fibers do not act as reinforcing agents [17,18]. The authors attributed this mainly to the poor interfacial adhesion between the polymeric matrix and hydrophilic lingo-cellulosic fillers which does not allow for efficient stress transfer between the two phases of the material. Increased percentages of fillers having high particle dimensions lead to the depreciation of mechanical properties, while smaller particle dimensions result in better mechanical properties due to higher total surface area of the filler particles that lead to a more efficient stress transfer mechanism than the larger particles [19,20,21].

The goal of the paper is to present the obtaining by electron beam cross-linking and characterization of a new type of eco-composite based on natural rubber and wood sawdust as filler (NR/wood sawdust). The influence of wood sawdust amount and electron beam irradiation dose on the physical and mechanical properties, cross-linking density rate and behavior in aqueous environment, was studied.

## 2. Experimental Section

### 2.1. Materials

The raw materials used in the experiments were: natural rubber of Crep 1× type (Mooney viscosity of 74 ML_1+4_ at 100 °C, volatile materials content of 0.32%, nitrogen content of 0.38%, percentage of ash of 0.22%, impurities content of 0.021%), pentaerythritol tetrakis 3-(3,5-di-tert-butyl-4-hydroxyphenyl) propionate Irganox 1010 as antioxidant, polyethylene glycol PEG 4000 (density of 1.128 g/cm^3^, melting point range between 4–8 °C), wood sawdust as filler. The used wood sawdust was beech wood obtained from a local sawmill in Romania. Its properties were constant, assured by the dimensions of particles (mesh 250–270) and purity (the wood sawdust was obtained by processing a single type of wood).

### 2.2. Sample Preparation

Blends were prepared on an electrically heated laboratory roller. For preparation of the polymeric composites the blends constituents were added in the following sequences and amounts: 100 parts natural rubber roll binding (2 min), embedding 3 phr (parts to 100 parts rubber) PEG 4000 and 1 phr Irganox 1010 antioxidant (2 min), adding 10 and 20 phr wood sawdust respectively (2–4 min), homogenization of blends and removing from the roll in the form of sheet (4 min). Process variables were as follows: temperature between 25–50 ± 5 °C, friction 1:1.1 and total blending time 8–14 min. Plates required for physical and mechanical tests with sizes of 150 × 150 × 2 mm^3^ were obtained by pressing in a hydraulic press at 110 ± 5 °C and 150 MPa.

### 2.3. Experimental Installation and Sample Irradiation

The samples prepared as was described above, were packed in polyethylene film and irradiated at 75, 150, 300 and 600 kGy, respectively, using the electron beam accelerator called ALID 7, in atmospheric conditions and at room temperature of 25 °C. ALID-7 was built in the Electron Accelerator Laboratory from the National Institute for Lasers, Plasma and Radiation Physics, Bucharest, Romania. ALID 7 is of travelling-wave type, operating at a wavelength of 10 cm. The accelerating structure is a disk-loaded tube operating in the *π*/2 mode. The optimum values of the electron beam, peak current I_EB_ and EB energy E_EB_ to produce maximum output power P_EB_ for a fixed pulse duration *τ*_EB_ and repetition frequency f_EB_ are as follows: E_EB_ = 5.5 MeV, I_EB_ = 130 mA, P_EB_ = 670 W (f_EB_ = 250 Hz, *τ*_EB_ = 3.75 µs). Radiation dosimetry was assured by using the PTW-UNIDOS high performance secondary standard dosimeter for universal use, connected to the Advanced Markus Electron Chamber that is a plane parallel ion chamber for high-energy electron measurements. The chamber features a flat energy response within the nominal energy range from 2 MeV to 45 MeV. It was placed under the accelerator exit window, in the middle of the electron beam cross section and the values obtained were read in the accelerator control room with the PTW-UNIDOS dosimeter, 10 s for each measurement and after that, an average dose rate was considered. An important step is the establishing of the electron beam penetration depth in sample, in order to ensure equal doses at the entry and at the exit of the irradiated sample. The thickness requirement of the material can be calculated from the following relation [22]:

(1) E=2.6⋅t⋅ρ+0.3

where *E* (MeV) is the beam energy, in our case 5.5 MeV, *t* (cm) is the thickness and *ρ* (g·cm^−3^) is the sample density, in our case 1 g·cm^−3^. The thickness of the irradiated samples was set to 20 mm.

### 2.4. Laboratory Tests

#### 2.4.1. Mechanical Characteristics

The tensile properties of samples were determined using a Schopper tensile tester with a nominal rate of the traverse of the moving grip of 460 mm/min. The tensile strength was determined according to ISO 37/2012, on dumbbell shaped specimens. The hardness, in units of Shore A, was measured according to ISO 7619-1/2011 using a hardness tester. The elasticity was evaluated according to ISO 4662/2009, with a Schob test apparatus using 6 mm thick samples. Test specimens were cut off from plates of 150 × 150 × 2 mm by means of an automatic punching die.

The sol-gel analysis was performed on cross-linked NR rubber, with and without wood sawdust used as filler in order to determine the gel fraction, i.e., the mass fraction of insoluble NR resulting from the network-forming cross-linking process. Samples having known mass were swollen in toluene for 72 h in order to remove any split fragments and unreacted materials and were dried in air for 6 days and then in a laboratory oven at 80 °C for 3 h. Finally, samples were re-weighed. The gel fraction was calculated as follows:

(2) $$Gelfraction=\frac{m_{s}}{m_{i}}\times 100$$

where $m_{s}$ and $m_{i}$ are the mass of the dried sample after extraction and the initial mass of the sample, respectively [23,24].

*The crosslink density* (ν) of the samples was determined on the basis of equilibrium solvent-swelling measurements (in toluene at 23–25 °C) by application of the well-known modified Flory-Rehner equation for tetra functional networks. The samples of 2 mm thickness were initially weighed (*m_i_*) and immersed in toluene for 72 h. The swollen samples were cautiously dried to remove the solvent excess and weighed (*m_g_*) being covered to avoid toluene evaporation during weighing. Traces of solvent and other small molecules were eliminated by drying in air for 6 days and then in an oven at 80 °C for 3 h. Finally, the samples were weighed for the last time (*m_s_*), and volume fractions of polymer in the samples at equilibrium swelling *ν*_2m_ were determined from swelling ratio *G* as follows:

(3) $$\nu_{2\text{m}}=\frac{1}{1+G}$$

where:

(4) $$G=\frac{m_{g}-m_{s}}{m_{s}}\times \frac{\rho_{e}}{\rho_{s}};$$

$\rho_{e}$ and $\rho_{s}$ are the densities of elastomer samples and solvent (0.866 g/cm^3^ for toluene), respectively.

The densities of elastomer samples were determined by hydrostatic weighing method, according to SR ISO 2781/2010. The cross-linking densities of samples, *ν*, were determined from measurements in a solvent, using the Flory–Rehner relationship:

(5) $$\nu =-\frac{Ln\left(1-\nu_{2\text{m}}\right)+\nu_{2\text{m}}+\chi_{12}\nu_{2\text{m}}^{2}}{V_{1}\left(\nu_{2\text{m}}^{1/3}-\frac{\nu_{2\text{m}}}{2}\right)}$$

where, $V_{1}$ is the molar volume of solvent (106.5 cm^3^/mol for toluene), $\nu_{2m}$ is the volume fraction of polymer in the sample at the equilibrium swelling, and $\chi_{12}$ is the Flory-Huggins polymer-solvent interaction term (the value of and $\chi_{12}$ is 0.393 for toluene) [23,24].

*Water uptake tests* were done in accordance with SR EN ISO 20344/2004 in order to study the effect of water absorption on fiber reinforced natural rubber composites. The samples were dried in a laboratory oven at 80 °C for 2 h and then were cooled at room temperature in desiccators before weighing. Water absorption tests were conducted by immersing the samples in distilled water in beaker and then maintaining at room temperature (23 ± 2 °C). After immersion, the samples were taken out from the water at periodic intervals and the wet surfaces were quickly dried using a clean dry cloth or tissue paper before weighing. The moisture absorption was calculated from the weight difference. The percentage of weight gain of the samples was measured at different time intervals. The water uptake was calculated as follows:

(6) $$\text{Water uptake }(\%)=\frac{W_{S}-W_{1}}{W_{1}}\times 100$$

where, *W_S_* is the weight of the sample saturated with water, determined at periodic intervals and *W*_1_ is the initial weight of the oven-dried specimen.

#### 2.4.2. Rubber-Filler Interactions

The extent of interaction between rubber and filler (wood sawdust) can be analyzed using the Kraus equation. The Kraus theory and Kraus equation [25] have been successfully used by some researchers to assess the interfacial interaction in filler-reinforced rubber composites [26,27]. The Kraus equation is expressed as follows:

(7) $$V_{ro}/V_{rf}=1-m\frac{f}{1-f}$$

where, *V_ro_* and *V_rf_* are the volume fractions of the rubber in the vulcanized gum and in the filled swollen sample respectively, *f* is the volume fraction of filler and *m* the filler polymer interaction parameter. The volume fraction of rubber in the swollen sample *V_rf_*, was calculated using the following expression:

(8) $$V_{rf}=\frac{\left[\left(D-FT\right)/\rho_{r}\right]}{\left[\left(D-FT\right)/\rho_{r}\right]+\left(A_{0}/\rho_{S}\right)}$$

where $\rho_{r}$ and $\rho_{s}$ are the densities of rubber samples and solvent (0.94–1.0 g/cm^3^ for natural rubber and 0.866 g/cm^3^ for toluene) respectively, *D* is the de-swollen weight of the test specimen (dry weight), *F* is the weight fraction of the insoluble components, *T* is the weight of the specimen and *A*_0_ the weight of the absorbed solvent at equilibrium swelling.

#### 2.4.3. Fourier Transform Infrared Spectroscopy (FTIR)

The changes in chemical structure of the composites based on NR and wood sawdust were highlighted using a FTIR spectrophotometer—TENSOR 27 (Bruker, Bremen, Germany) by ATR measurement method. The samples spectra were obtained from 30 scans mediation, realized in absorption in the range of 4000–600 cm^−1^, with a resolution of 4 cm^−1^.

#### 2.4.4. Scanning Electron Microscopy (SEM)

The surface texture of the composites based on NR and wood sawdust was examined using the scanning electron microscope FEI/Phillips (FEI Company, Hillsboro, OR, USA). All the surfaces were placed on an aluminum mount, sputtered with gold palladium and then scanned at an accelerating voltage of 30 kV.

## 3. Results and Discussion

### 3.1. Physical and Mechanical Characteristics

Physical and mechanical characteristics of the composites based on NR and wood sawdust (NR/wood sawdust), obtained by electron beam cross-linking are presented in Figure 1, Figure 2, Figure 3, Figure 4, Figure 5, Figure 6, Figure 7 and Figure 8.

**Figure 1:**
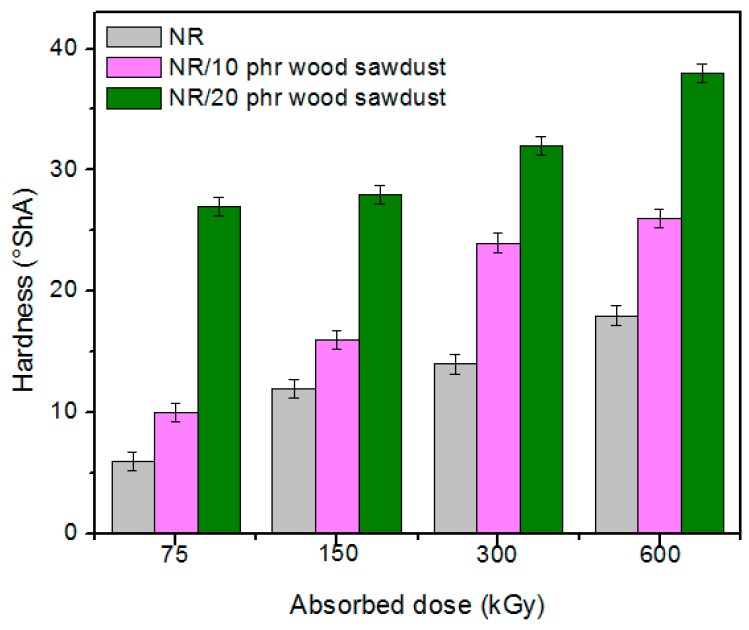
Hardness variation as a function of wood sawdust amount and irradiation dose.

**Figure 2:**
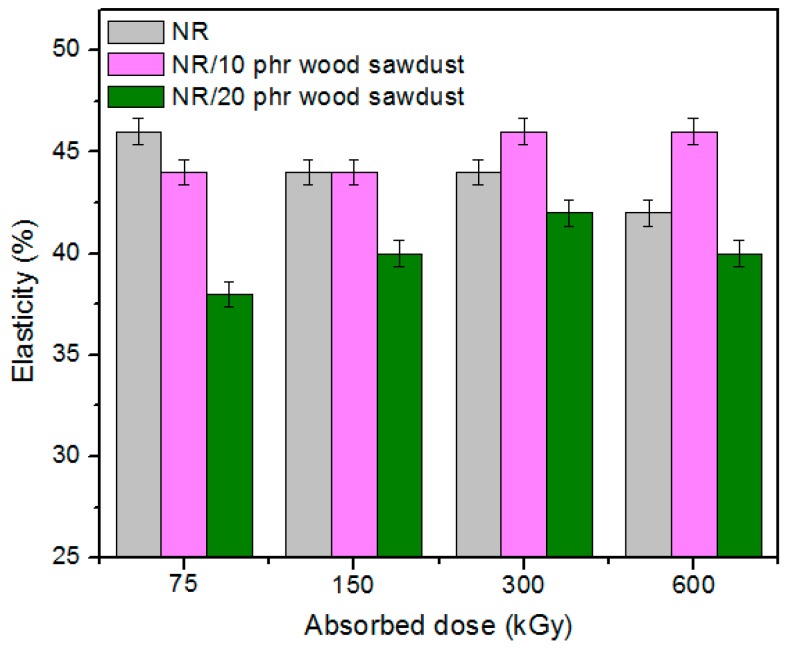
Elasticity variation as a function of wood sawdust amount and irradiation dose.

**Figure 3:**
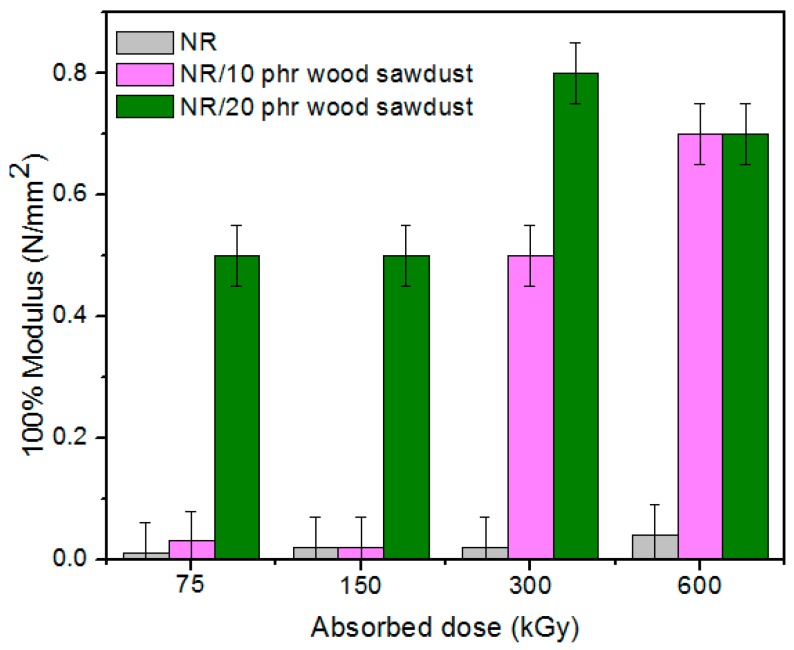
100% Modulus variation as a function of wood sawdust amount and irradiation dose.

**Figure 4:**
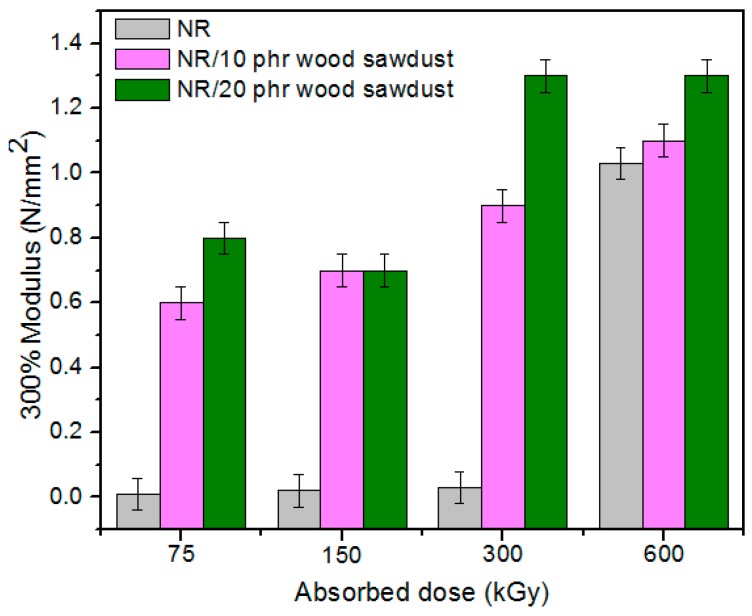
300% Modulus variation as a function of wood sawdust amount and irradiation dose.

**Figure 5:**
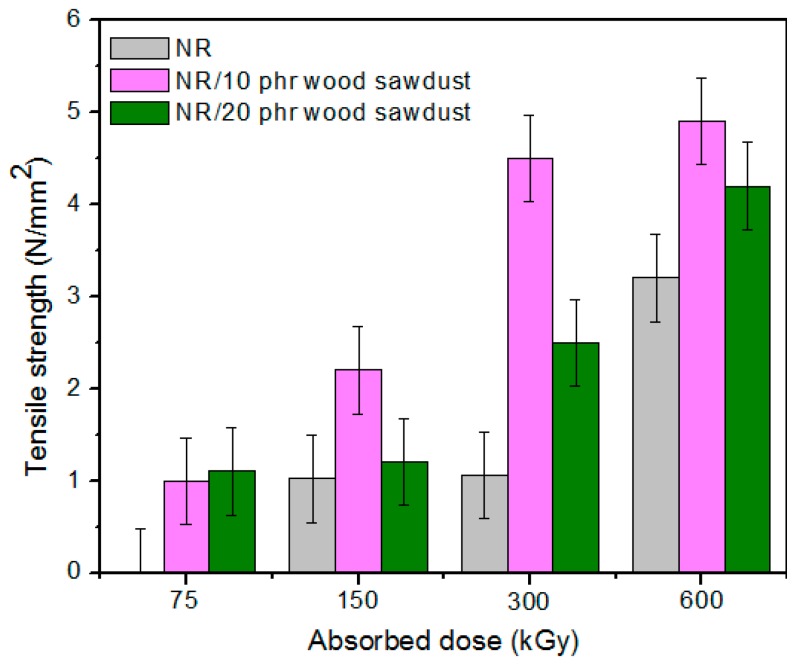
Tensile strength variation as a function of wood sawdust amount and irradiation dose.

**Figure 6:**
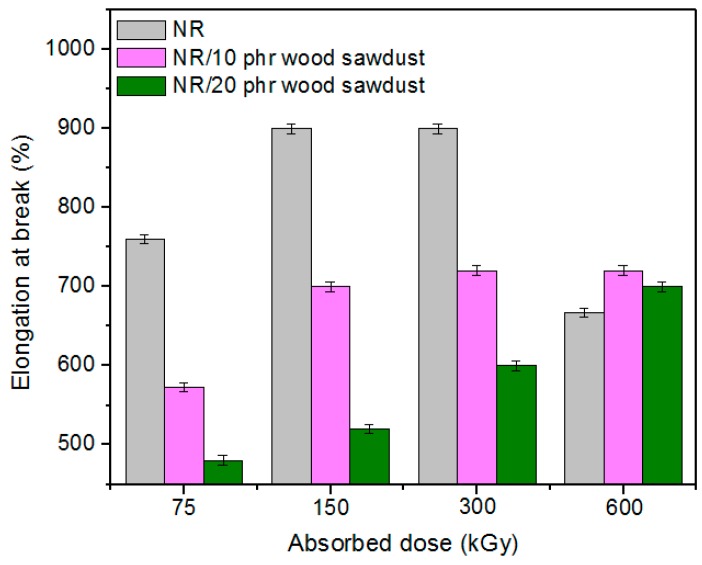
Elongation at break variation as a function of wood sawdust amount and irradiation dose.

**Figure 7:**
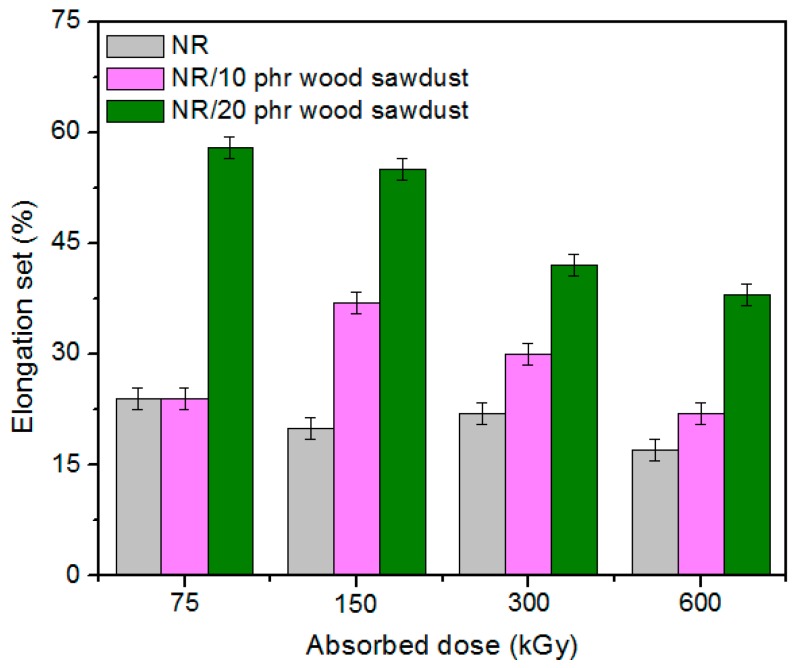
Elongation set variation as a function of wood sawdust amount and irradiation dose.

**Figure 8:**
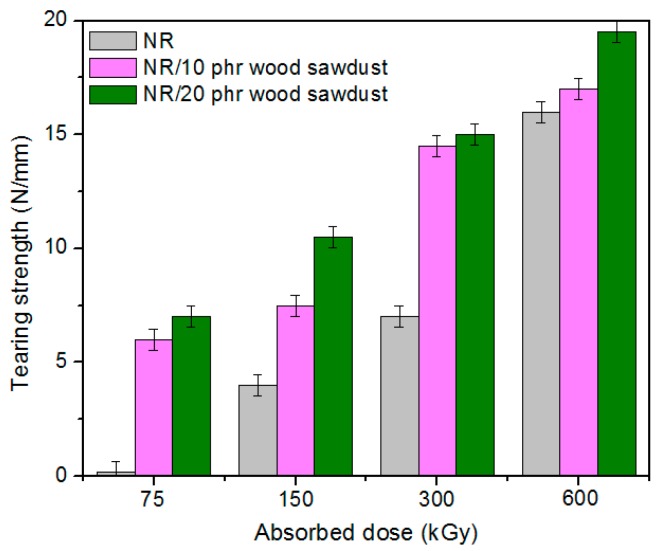
Tearing strength variation as a function of wood sawdust amount and irradiation dose.

From the above figures, it can be seen that mechanical properties of the NR/wood sawdust composites cross-linked by EB irradiation were noticeably affected by increasing the wood sawdust concentration, compared with the NR samples. Except the elasticity and elongation set, all studied properties were improved by the addition of wood sawdust. Hardness is the measure of how resistant is a solid material when a force is applied. From Figure 1, it is observed that hardness has increased with both irradiation dose and wood sawdust amount in blend. This happened because the wood sawdust incorporated into the NR matrix has conducted in a reduction of plasticity and flexibility of the rubber chains, so the composite has become more rigid [28]. The hardness increase can also be due to the reinforcement effect of the filler as well as to the extent of cross-linking in the polymeric material [28]. One-hundred percent Modulus, 300% Modulus, tearing and tensile strengths have increased whit the increasing of the absorbed dose and the introducing of wood sawdust in natural rubber blends. This can be attributed to the occurrence of a strong interface as well as to the close packing arrangement in the composite. Tensile strength is the maximum force to which a material can withstand without fracturing when stretched and is an indication of how strong a compound is. Maximum elongation is the measure of how much a specimen stretches before it breaks**.** As expected, the incorporation of wood sawdust in the matrix has improved the tensile strength (Figure 5), due to the strong interaction between the natural rubber and filler which effectively has constrained the motion of polymer chains [29,30], due to the good adhesion of the filler in matrix and because of the agglomeration of filler particles [27,28]. Therefore, the surface reactivity which has determined the polymer–filler interaction, the aggregates, sizes and shapes of fillers and finally, the structural and filler particle dispersions in rubber had improved the modulus [28]. Modulus is the stress required to produce a certain elongation (strain). This elongation might be 50%, 100%, or even 300%, though 100% is the most widely used for testing and comparison purposes. In our experiments, we tested only 100% Modulus or 300% Modulus, that means 100% Elongation or 300% Elongation. Because it is basically a measure of tensile strength at a particular elongation (rather than at rupture), modulus is also known as tensile modulus or tensile stress. Modulus is typically gauged simultaneously with tensile strength on the same specimen. In addition, 100% and 300% Modulus are in close correlation with the degree of crosslinking of the materials, as meaning that a higher crosslinking degree leads to a higher Modulus. For NR, the crosslinking degree shows a significant increase at 300 to 600 kGy absorbed dose, and this explains the significant increase of 100% and 300% Modulus at these doses, as shown in Figure 3 and Figure 4. In addition, in these figures can be seen that 100% and 300% Modulus increases with the increase of wood sawdust content in the composites. Usually, the modulus is related to the stiffness of the rubber. Therefore, the increase in wood sawdust amount enhances the stiffness, which may be the cause of increasing the modulus of the composite materials [31]. As seen in Figure 1, the hardness has increased with irradiation dose increases and wood sawdust amount in blend. Therefore, the composite has become more rigid and the 100% and 300% Modulus increases**.** The increasing of 100% and 300% modulus and elasticity, with increasing of the wood sawdust amount is the indication that the filler had a good interaction with the rubber. Tearing strength, also named tear resistance, is the measurement of a sample’s ability to resist tearing. Tear strength is usually the maximum load divided by the thickness of the material. In Figure 8 is observed that the tearing strength has followed the same trend of growth with the fiber content in composite, as the hardness and tensile strength. All these results indicate that the wood sawdust has a reinforcing effect on natural rubber [13]. Elongation at break is the ratio between changed length and initial length after breakage of the test specimen. It expresses the capability of a material to resist changes of shape without crack formation. The elongation at break is the deformation (strain) on a sample when it breaks. Elongation at break (Figure 6) has decreased with the increase of EB dose and with the increase of wood sawdust amount introduced in blends, indicating an increase in cross-link density. The highlighted decrease is the result of the high cross-linking degree. More than that, its reduction indicates that the addition of wood sawdust into NR composites had a negative effect upon the ductility because of the appearance of a restriction in the molecular chains movement [32]. In addition, the striking forces between the filler and the polymer molecules, had led to the development of a cross-linked structure which limit the free mobility of the polymer chains, hence increases the resistance to accelerate upon the execution of tension [32,33]. Tensile set testing of a rubber evaluates the residual elongation and is named elongation set of a test sample after being stretched and allowed to relax in the specified manner. Tensile set is expressed as a percentage of the original length. The elongation set (Figure 7) has decreased with the increase of EB dose and with the increase of wood sawdust amount introduced in blends, indicating an increase in cross-link density. The decrease in residual elongation shows that the sample is vulcanized and thus returns to its original shape easily. Elasticity or rebound resilience (Figure 2) has varied irregular whit the increasing of the wood sawdust amount and with the EB dose. Elasticity is the ability of a material to return to its original shape after it has been stretched by applying a force, which is known as stress. There is a limit of the amount of stress that can be applied to a material before it reaches its ‘elastic limit’ and it deforms irreversibly. The decrease in the resilience may be explained by the wood sawdust particles action that introduces a mechanism by which the strain energy diminishes. The presence of the wood sawdust reduces the degree of elasticity and segment mobility of the cured NR composites, since the rebound resilience is directly proportional to them. The increasing of the amounts of filler, leads to the increasing of hardness and decreased elasticity (resilience) [28]. The mobility of wood sawdust particles and slippage of chains attributed to applied stresses on the cured composite increases its hysteretic behavior [34]. Therefore, the resilience decreases with increasing load of wood sawdust.

### 3.2. Gel Fraction and Crosslink Density of the Blends

**Figure 9:**
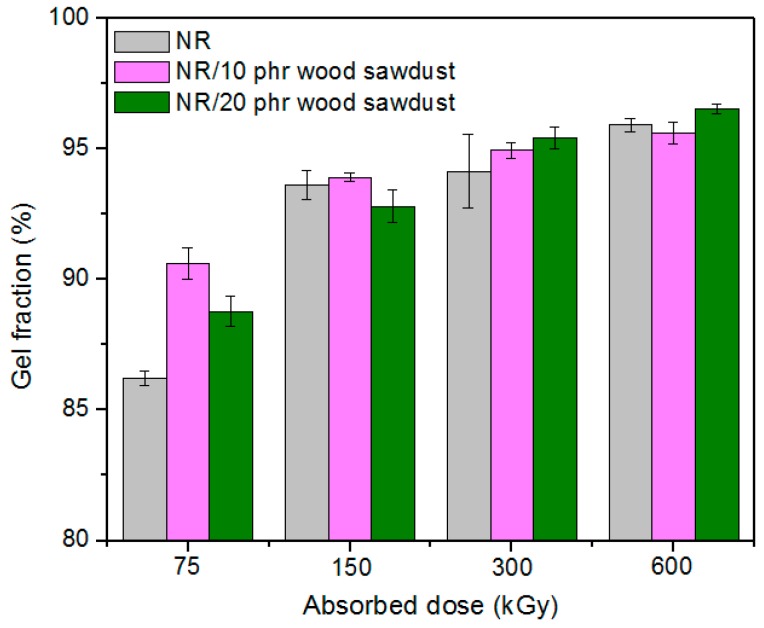
Effect of the absorbed dose and wood sawdust amount on the gel content (G).

**Figure 10:**
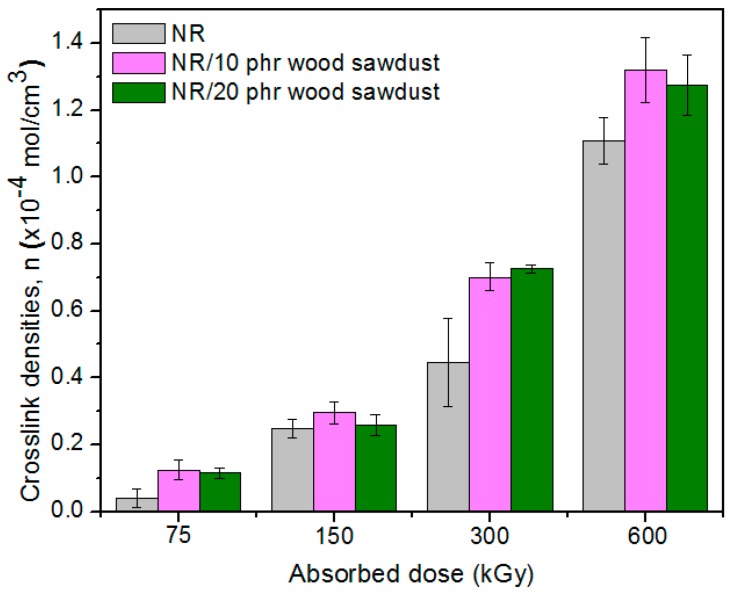
Effect of the absorbed dose and wood sawdust amount on the cross-link density (*ν*).

Figure 9 and Figure 10 shows the variations of the gel content (G) and cross-link density (*ν*) of the samples vulcanized by EB as a function of the wood sawdust content and absorbed dose.

**Figure 11:**
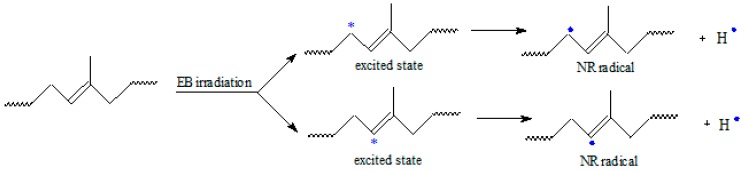
A possible mechanism of radicals formation in NR by electron beam irradiation.

The gel fraction values, except for the samples of blend natural rubber without sawdust and irradiated with 75 kGy, are over 90% for all blends and varies irregular depending on the amount of wood sawdust in the blend, the cross-linking method and the irradiation dose. As the wood sawdust quantity in blends increases, the cross-link density (*ν*) increases also, because the filler action of the wood sawdust in natural rubber blends, leads to their reinforcement. The effects of ionizing radiation on rubbers consist in the occurrence of cross-linking and degradation effects. These reactions are reported to follow the free radical mechanism. The elastomers cross-linking by means of EB is done without heating and without vulcanization agents. The reaction mechanism is similar to the cross-linking by means of peroxides, but in this case, as is shown in Figure 11, the reaction initiation is due to the EB action. The ionizing radiation produces the excitation of polymer molecules and the energies associated with the excitation depend on the absorbed dose. Free radicals, that can react directly by connecting the polymer chains or can initiate grafting reactions, are formed [1,3,10].

**Figure 12:**
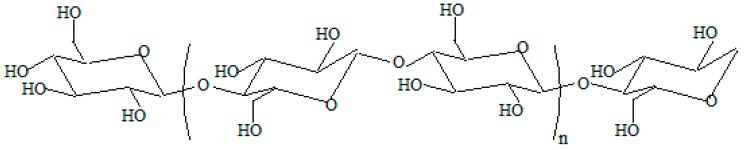
The structure of cellulose.

The wood sawdust consists mainly of the following structures: cellulose (42%–49%), hemicellulose (23%–34%), lignin (20%–26%), extractives (3%–8%) and ash (0.2%–0.8%). Chemically, cellulose is referred to as linear (1 → 4)-*β*-d-glucan [35,36]. Glucan molecule is a polysaccharide of d-glucose monomers linked by glycosidic bonds. In Figure 12 is presented the structural formula of cellulose, in which ^4^C_1_ is the most stable conformation.

**Figure 13:**
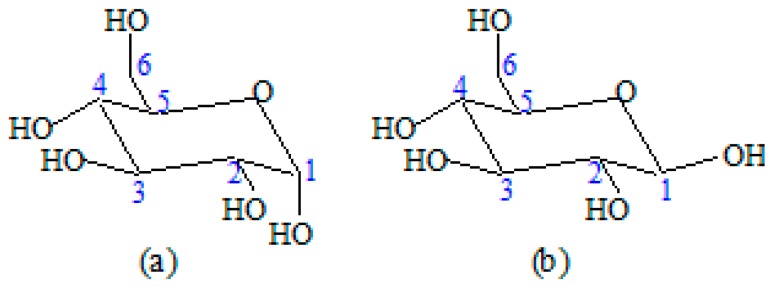
The representation of (**a**) *α*-d-glucopyranose and (**b**) *β*-d-glucopyranose in the ^4^C_1_ conformation.

In Figure 13 can be seen that the molecule takes the shape of a chair with C(4) above the plane of the ring (formed by C(2), C(3), C(5) and the oxygen ring) and C(1) below the plane of the ring [36,37]. In addition, when the nucleophilic addition takes place, two possibilities arise for the carbonyl group: the resulting hydroxyl group (at C(1) thus) can orient itself *cis* or *trans* with the hydroxyl group on C(4) which corresponds in the case of d-glucopyranose with *α*-d-glucopyranose respectively *β-*d-glucopyranose [35,36]. In both crystalline and non-crystalline structures, cellulose is found. The cellulose can obtain a crystalline structure by the coalescence of several polymer chains which leads to the formation of micro-fibrils which themselves are united to form fibers [38].

**Figure 14:**
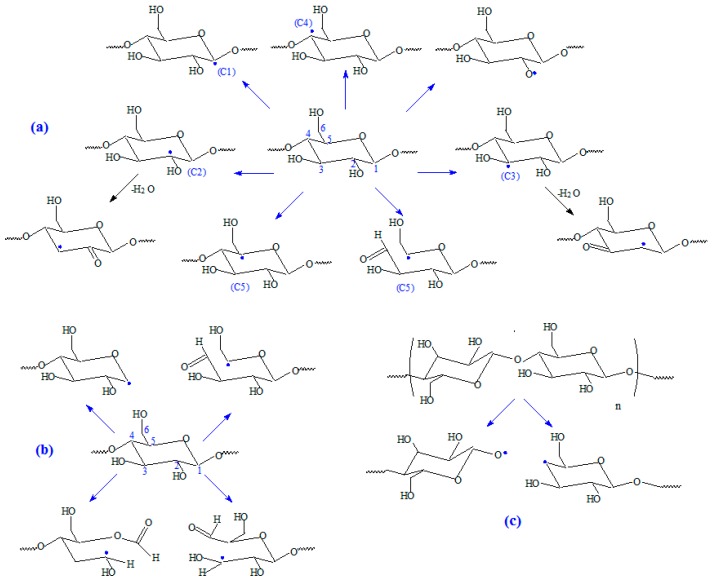
Possible structures of radicals formed by hydrogen abstraction in cellulose by electron beam irradiation. (**a**) Formation of radicals with localized unpaired electrons; (**b**) Formation of radical species by cleavage; (**c**) Formation of radical species by chain scission.

Hemicellulose is a branched polysaccharide polymer built of xylan, glucomannans, glactoglykomannan, arabinogalacan and galactan, which exist in an amorphous form and which is not stable to chemical attacks as cellulose is [39]. Lignin is the most complex natural polymer, an amorphous three-dimensional polymer having phenylpropane units as predominant building blocks. More specifically, p-coumaryl alcohol, coniferyl alcohol and sinapyl alcohol are the ones most commonly encountered [38]. Extractives are mainly resins and fatty acids, resin acids and esters [39]. The polymer composites reinforced with natural fibers present a poor bonding between the cellulose fiber and the polymer matrix due to their opposite chemical nature [40]. This it was overcome by chemical or physical processing of the natural fibers [40,41,42]. The chemical processes were developed to clean the fiber surface, to modify the surface chemically, to restrain moisture absorption and to make the surface more hydrophobic and thereby to remove the hydrophilic groups of natural fibers. Physical treatments such as plasma treatment, corona treatment or electron beam irradiation have been conducted in order to create a hydrophobic group, to cause cross-linking and to increase the interfacial surface area [40,43,44]. There are several studies in which the effects of electron beam irradiation on cellulose have been evaluated [45,46]. More than 20 different cellulose macro-radicals, including primary and secondary species, can be formed and distinguished. Some of them are thermodynamically favored and thus far more likely to be created [36,47,48]. During irradiation of cellulose, radicals with localized unpaired electrons are formed, mainly in positions 1, 2, 3, 4 and 5 of the pyranose ring, as is showed in Figure 14a. These radicals should originate from hydrogen abstraction from positions mentioned above and from the OH group [49]. The radicals formed from the positions 2 and 3 of the pyranose ring may form other radicals by loss of a water molecule as it can be seen in Figure 14a. On the other hand radical species can be formed by cleavage of a glycosidic bond and from *β*-fragmentation of an oxygen-centered radical resulting from cleavage of a glycosidic bond [49] and by chain scission, as shown in Figure 14b,c. According to the mechanisms presented in Figure 14a–c it can be seen that free radicals are formed after C–H, C–O and C–C bond cleavages by hydrogen abstraction, chain scission and cycle opening.

**Figure 15:**
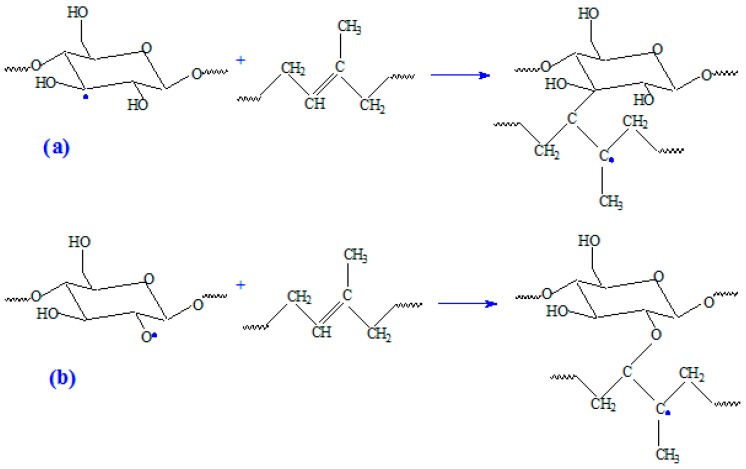
Possible cross-linking mechanism between cellulose from wood sawdust and natural rubber molecules through: (**a**) C–C bond and (**b**) C–O–C bond.

In our study, wood sawdust fibers with high cellulose content are used as fillers in a natural rubber matrix. By EB irradiation, cross-linking, grafting and degradation of these types of NR/wood sawdust composites mainly occurs. The cross-linking process leads to the increase of composites cross-linking degree and to the improvement of some physical and mechanical properties. The grafting of NR macromolecules leads to the formation of a grafted co-polymer at the interface between the two phases, a fact that will significantly improve their compatibility leading to the obtaining a polymeric composite having optimum properties. Natural rubber and cellulose from wood sawdust join together through the C–C and C–O–C bonds, as shown in Figure 15. Our experimental results, confirmed by other author studies [27,28,32], show that with the increase of cross-link density, the hardness [28,29] and tensile strength [27] have increased, whereas the elongation-at-break decreased [32]. Thus, it can be considered once again that wood sawdust acted as filler in natural rubber blends and leads to reinforcement of them.

### 3.3. The Water Uptake

**Figure 16:**
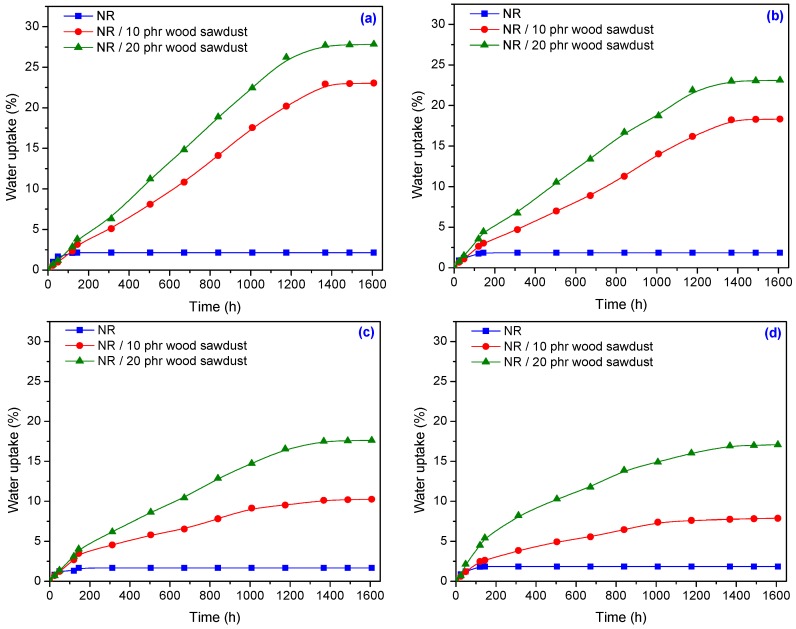
The water uptake according to the amount of wood sawdust and irradiation dose at 23 ± 2 °C: (**a**) 75 kGy; (**b**) 150 kGy; (**c**) 300 kGy; (**d**) 600 kGy.

**Figure 17:**
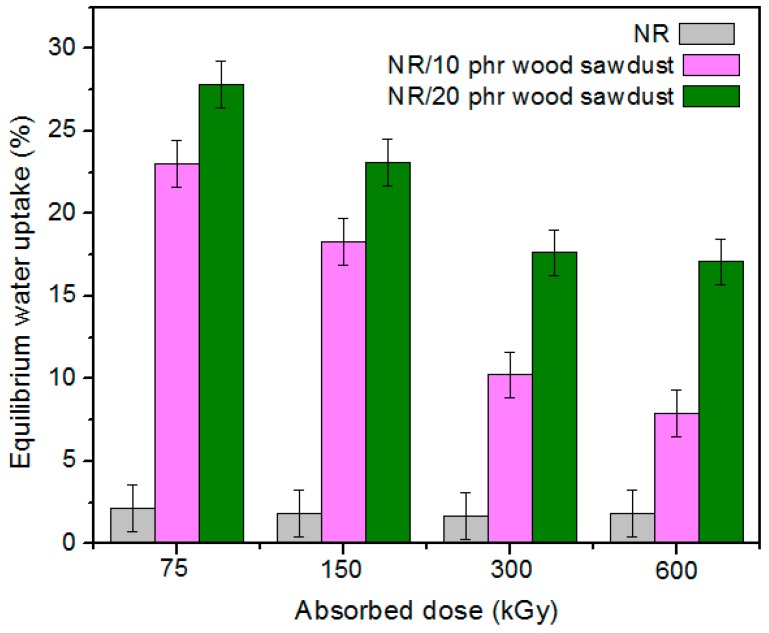
The water uptake of NR and NR/wood sawdust composites, according to the amount of wood sawdust and irradiation dose at 23 ± 2 °C.

The results of water uptake experiments made on samples with and without wood sawdust are presented in Figure 16. It is observed that the percentages of water absorption in the composites NR/wood sawdust, depends on two parameters: wood sawdust content and irradiation dose. The water uptake increased with increasing of wood sawdust content and decreased with increasing of absorbed dose. The increasing of water absorption is due to the hydrophilic nature of wood sawdust and to the bigger interfacial area between the wood sawdust and the elastomer matrix. In polymer composites having wood sawdust, water is absorbed mainly by the wood sawdust because the rubber material is hydrophobic and its water absorbability can be neglected [50]. The hydrophilic nature of wood sawdust may be a major problem, as in the case of all cellulose-fibers, if it is used as reinforcement in plastics or rubbers. The moisture content is dependent on the non-crystalline parts and voids. From Figure 16 it is observed that the water absorption of composites has increased with the increasing of filler amount, for all irradiation doses. With the increasing of wood sawdust content in the composite, the number of free –OH groups derived from cellulose and hemicellulose, increases also. These free –OH or hydroxyl groups come in contact with water and form hydrogen bonds, which result in a weight gain in the composites [51]. However, the water uptake has decreased with increasing of absorbed dose, as shown in Figure 17. An explanation may be connected with the removal of some –OH groups inside the cellulose, which results in a reduction of free –OH groups inside the entire composite, so a low availability to absorb water [51].

In addition, responsible for these results may be also the PEG presence in the composition of elastomeric matrix, because several studies have shown that PEG may be used for the improvement of the interactions at the fiber/matrix interface, because acts not only as a plasticizer for rubber, but also as compatibilizer between the hydrophobic rubber and the hydrophilic fibers. It is also well known that PEG prevents the aggregation of the fibers, so that the filler is homogeneously dispersed in the rubber matrix and form a network structure. On the other hand, it has been shown that the PEG has improved the intermolecular interactions, because of the existence of intermolecular hydrogen bonds among the rubber matrix, PEG and natural fibers [52,53,54].

### 3.4. Rubber-Fiber Interactions

The extent of interaction between rubber and filler it was analyzed using Kraus equation and the results are listed in Table 1.

The results presented in Table 1 show that the equilibrium solvent uptake of the samples decreased when the fiber content increased for all absorbed dose, which caused an increase in *V_rf_*. Therefore, the ratio *V_ro_*/*V_rf_* decreases since *V_ro_* is a constant. This is due to the increased hindrance exerted by the wood sawdust filler at higher loadings. The diffusion mechanism in the composite is strongly connected with the ability of rubber to provide pathways for the solvent to progress in the form of randomly generated voids. As the void formation decreases with the filler content, the solvent uptake also decreases. The ratio *V_ro_*/*V_rf_* is the degree of restriction of swelling of the rubber matrix due to the presence of fillers [27,55]. The more and more reduced values of *V_ro_*/*V_rf_* ratio are associated with the enhanced adhesion between filler and rubber, according to the Kraus theory and Kraus equation. The decreased values of *V_ro_*/*V_rf_* at higher loadings indicate the reinforcement effect of the wood sawdust.

### 3.5. The Effect of Electron Beam Radiation on NR/Wood Sawdust Composites

**Figure 18:**
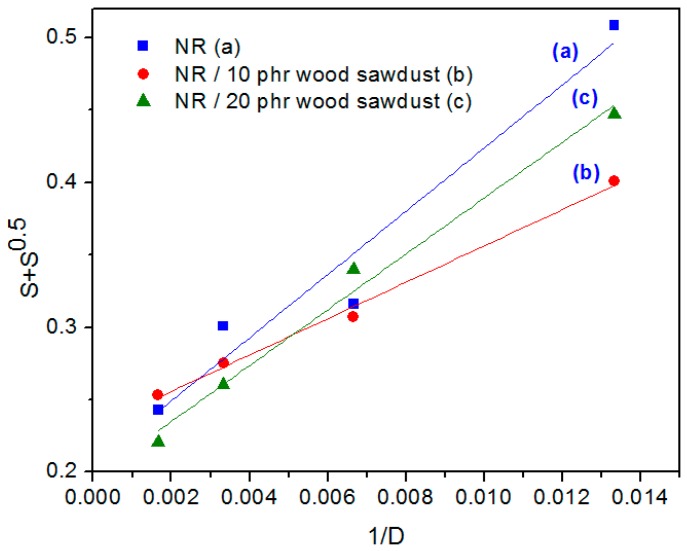
Charlesby-Pinner plot for NR/wood sawdust composites.

In order to quantitatively evaluate the yields of cross-linking and chain scission of the NR/wood sawdust composites irradiated with EB, were drawn the plots of S + S^1/2^ vs. 1/absorbed dose (D) from the Charlesby-Pinner equation for the different blend compositions (Figure 18) [55,56]:

(9) $$S+\sqrt{S}=\frac{p_{0}}{q_{0}}+\frac{1}{\alpha P_{n}D}$$

where, *S* is the sol fraction (*S* = 1 − gel fraction), *p*_0_ is the degradation density, average number of main chain scissions per monomer unit and per unit dose, *q*_0_ is the cross-linking density, proportion of monomer units cross-linked per unit dose, *P_n_* is the number averaged degree of polymerization, and *D* is the radiation dose in Gy.

In Table 2, the compositional characteristics, designation and *p*_0_/*q*_0_ for NR/wood sawdust blends are presented.

From Table 2 and Figure 18 it is observed that the sample of NR having 20 phr of wood sawdust is the most effective cross-linked by EB irradiation. The cross-linking extent increases almost linearly with wood sawdust content in the composite. Low values of *p*_0_/*q*_0_ for high wood sawdust content are suggestive for the relatively improved radical-radical interactions in polymer composite, probably due to the decrease in free-volume [55,57,58].

### 3.6. Fourier Transform Infrared Spectroscopy (FTIR)

**Figure 19:**
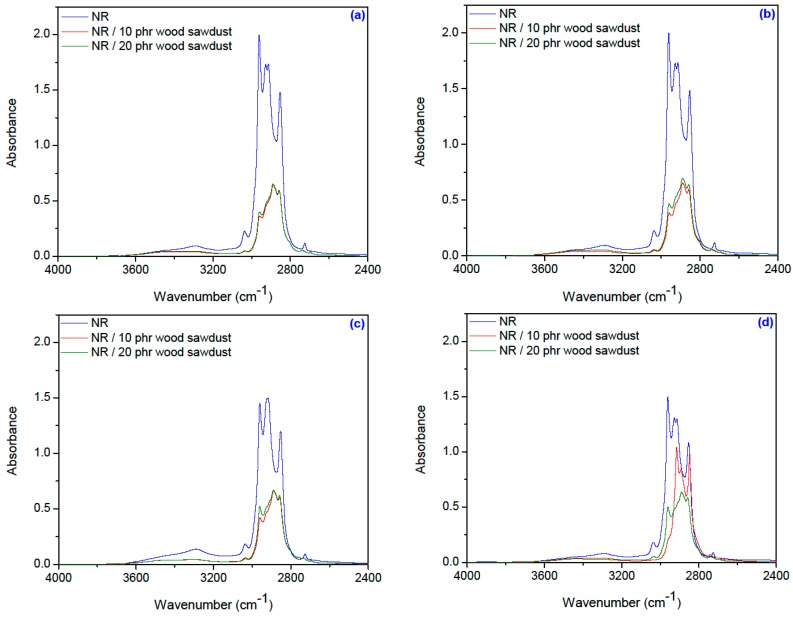
FTIR spectra for NR and NR/wood sawdust composite in the range of 4000–2400 cm^−1^ (**a**) 75 kGy; (**b**) 150 kGy; (**c**) 300 kGy; (**d**) 600 kGy.

**Figure 20:**
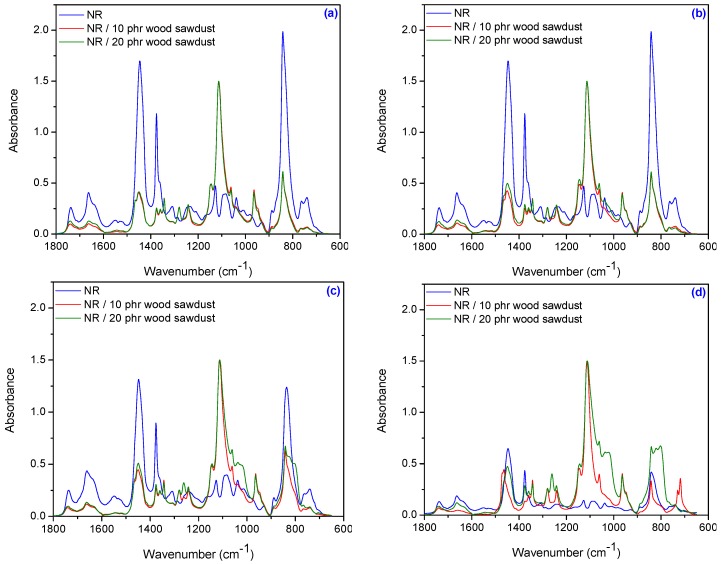
FTIR spectra for NR and NR/wood sawdust composite in the range of 1800–600 cm^−1^ (**a**) 75 kGy; (**b**) 150 kGy; (**c**) 300 kGy; (**d**) 600 kGy.

Figure 19a–d and Figure 20a–d show the infrared spectra made in the range of 4000–650 cm^−1^ on samples of natural rubber and natural rubber/wood sawdust composites vulcanized with electron beam.

The natural rubber is composed of hydrocarbons (89.3–92.4 wt. %), proteins (2.5–3.5 wt. %), and other ingredients (4.1–8.2 wt. %). The main component of natural rubber is *cis*-1,4-polyisoprene, having a long chain and a high degree of branching. The main components of natural fibers are cellulose (*α*-cellulose), hemicellulose, lignin, pectins and waxes [59] and the first three represent 99% of the wood sawdust’s composition. The bonding process between the matrix (natural rubber) and the filler (wood sawdust) is due to cellulose radicals formation with localized unpaired electrons mainly in positions 1, 2, 3, 4 and 5 of -the pyranose ring, during the irradiation (Figure 14). On the other hand, the sawdust contains polymers cellulose, hemicelluloses and lignin, which possess many active functional groups susceptible to chemical reactions, such as: primary and secondary hydroxyls, carbonyls, carboxyls, esters, ether etc. [60].

Absorption bands with maxima at 3035–3036 cm^−1^ corresponding to CH stretching in the –CH=CH_2_ group, are observed in Figure 19. In Figure 19a is observed a decrease in intensity for this band, as follows: from 0.224 for NR to 0.045 and 0.048 for NR with 10 and 20 phr wood sawdust respectively, at the lowest irradiation dose: 75 kGy. With irradiation dose increasing, the intensity of this band for samples with NR falls very little and equals values for NR with wood sawdust. Thus, for 600 kGy dose (Figure 19d), the intensity is 0.181 for NR and 0.051 and 0.053 respectively, for NR with 10 and 20 phr wood sawdust. Vulcanization leads in the consumption of the double bonds of NR molecules, so that the intensities of these absorption bands decrease. The characteristic bands of the saturated aliphatic sp^3^ C–H bonds are observed in the region of 2970–2830 cm^−1^ which are assigned to *ν*_as_ (CH3), *ν*_as_ (CH2), and *ν*_s_ (CH2), respectively [61]. At dose of 75 kGy (Figure 19a) the intensity of these bands is big for NR and decrease with irradiation dose increase, so is seen in Figure 18b–d. At dose of 150 kGy (Figure 19b) the intensity of these bands is bigger for samples that contain wood sawdust, and at 300 kGy (Figure 19c) their intensity decrease for NR. The intensities of these bands equalize at the biggest irradiation dose, 600 kGy (Figure 19d). According to these results, the decrease in intensity of these bands with increasing radiation dose may be due to splitting these groups because, when materials are irradiated by ionizing radiation, two types of reactions occur, degradation (chain scission) and chain link (crosslinking).

The broad band in the 3600–3100 cm^−1^ region, which is due to the OH-stretching vibration, gives considerable information concerning the hydrogen bonds. The peaks characteristic of hydrogen bonds from the spectra of amorphous celluloses have lower intensity, which can be correlated with the scission of the intra- and intermolecular hydrogen bonds. The water molecule can penetrate only the amorphous region and get linked with the available hydroxyl group. The fact that the peaks characteristic of hydrogen bonds from the spectra of amorphous celluloses (around of 3340 cm^−1^) have lower intensity can be attributed to electron beam irradiation, which has reduced the OH group, as well as has increased crystalline regions through cross-linking. This can explain the decrease of water uptake for NR/wood sawdust composites by increase of absorbed dose [62,63].

The presence of amorphous cellulose can be confirmed by the band from 2940–2840 cm^−1^, corresponding to the C–H stretching vibration in methyl and methylene groups [63]. These bands are specific to natural rubber and cellulose, lignin or hemicellulose, from the wood sawdust fibers existing in the mixture [64]. Cellulosic materials consist of crystalline and amorphous domains, in varying proportions, depending on both source and history. The physical properties of cellulose, as well as their chemical behavior and reactivity, are strongly influenced by the arrangement of the cellulose molecules with respect to each other and to the fiber axis, as well. Most of the reactants penetrate only the amorphous regions and it is only in these regions with a low level of order and on the surface of the crystallites where the reactions can take place, leaving the intracrystalline regions unaffected [63]. In addition, the absorption bands at 2970–2920 cm^−1^ indicate the presence of metoxy group (–OCH_3_) characteristic to lignin structure, respectively to the wood sawdust [60]. For NR samples with 10 and 20 phr wood sawdust, the intensities of these bands varies very little with irradiation dose increase (as is seen in Figure 19a–d), which means that the splitting of these groups from cellulose does not occur.

The absorption located in the region of 1740–1730 cm^−1^ corresponds to the >C=O stretch in non-conjugated ketones, carbonyls and in ester groups from NR [63], because it is known that the NR contains also other compounds, such as lipids, neutral glycolipids or phospholipids (Figure 20). In addition, wood sawdust clearly shows the absorption bands in the region of 1735 cm^−1^ due to >C=O stretching vibration. As seen in Figure 20a–d, when the irradiation dose increases, the carbonyl peak C=O at 1740 cm^−1^ was slightly shifted towards 1737 cm^−1^ because the ester carbonyl bonds was broken due to the radiation effect [51]. The presence of absorption bands in the region located between 1665–1660 cm^−1^, is due to valence vibration of homogeneous double bonds (C=C) in the NR structure. It is observed that their intensity decreases with absorbed dose increasing, due to the reduced amount of C=C. Approximately in the same region, 1635–1625 cm^−1^, the appeared absorption bands are due to the absorbed water in cellulose [65] or are caused by lignin aromatic skeletal vibrations [63]. Absorption bands located at 1510–1600 cm^−1^ are characteristic to the aromatic nuclei from wood sawdust [60]. In the region of 1480–1430 cm^−1^, the absorption known as “crystallinity band” [66] is due to both components from composite materials: on the one hand due to hydroxil (–OH) and methylene (–CH_2_–) groups from aromatic nuclei from wood sawdust and on the other hand to the methylene (–CH_2_–) groups from NR. As seen in Figure 20a–d, when the irradiation dose increases, the intensity of these peaks is significantly reduced for NR and is slightly increased and shifted for NR/wood sawdust, the most at 600 kGy absorbed dose (Figure 20d) due to the esterification of cellulose which corresponds to the decrease of hydrogen bonds [67]. More than that, methyl groups produce two bending bands, i.e., a symmetrical band at 1380 cm^−1^ and an asymmetrical band at 1475 cm^−1^. Methylene groups give rise to four types of bending vibrations: scissoring (1465 cm^−1^), rocking (720 cm^−1^), wagging (1305 cm^−1^) and twisting (1300 cm^−1^) [68]. The absorption band at 1380–1325 cm^−1^ is assigned to the asymetric and symmetric deformations of CH_3_ from NR [69]. The characteristic bands which appeared in the ranges of 1320–1305 cm^−1^ and 1290–1209 cm^−1^ are assigned to the cellulose component from wood sawdust (the bending vibration of C–H and C–O groups from aromatic rings) [63,65] and to the CH and CH_2_ groups from NR (CH in plane and to CH_2_ twisting and wagging deformation) [69]. All these bands in the ranges of 1380–1305 cm^−1^ do not present significant variations with irradiation dose increase or wood sawdust concentration. In Figure 20d, is observed an increase in intensity for the band from 1262 cm^−1^, at 600 kGy absorbed dose, attributed to the stretching vibrations of C–O groups. This kind of group is present in lignin and hemicelluloses structures and confirms the presence of wood sawdust in composite material. In the region of 1200–1100 cm^−1^, the absorption bands are due to the C–C stretching from NR and to the presence of the alcoholic hydroxyl (1150–1170 cm^−1^) and of the phenolic hydroxyl (1240–1265 cm^−1^) from wood sawdust [60,69]. The absorption located in the region of 1165–1150 cm^−1^ corresponds to the anti-symmetrical deformation of the C–O–C bond and the absorption located in the region of 1070–1020 cm^−1^ is due to the C–O, alcohol, O-H or aliphatic ethers [63,65]. In Figure 20c,d, the band at 1160 cm^−1^ which can be related to the antisymmetric stretching of C–O–C bond from wood sawdust (glycoside bonds), may indicate the formation of this bond with NR molecules. In addition, the band at 1038 cm^−1^ (associated with C–O stretching from cellulose and C–O deformation from primary alcohols of lignin) is increased and shifted that confirms the presence of wood sawdust in composite material obtained at high absorbed dose (300 and 600 kGy) [67]. The specific absorption bands of R_2_C=CH–R groups are observed between 836–838 cm^−1^. These changes occur as a result of the elastomer crosslinking and of the consuming of double bonds. The absorption bands between 628 and 820 cm^−1^ certify the presence of aromatic nuclei from sawdust [60] and these bands have high intensities for composites obtained at 600 kGy (Figure 20d).

### 3.7. Scanning Electron Microscopy (SEM)

**Figure 21:**
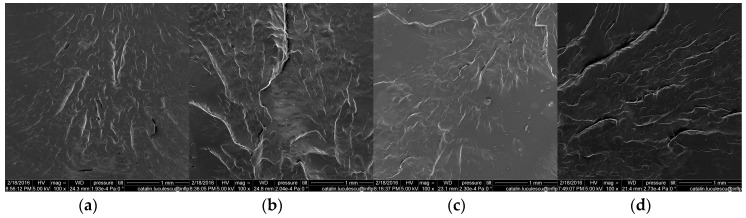
Scanning electron micrographs of the pure NR irradiated with (**a**) 75 kGy; (**b**) 150 kGy; (**c**) 300 kGy; (**d**) 600 kGy.

**Figure 22:**
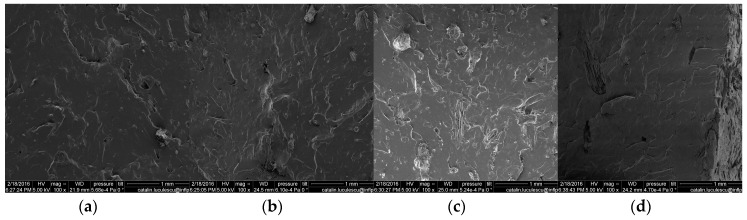
Scanning electron micrographs of the composites NR/10 phr wood sawdust, irradiated with (**a**) 75 kGy; (**b**) 150 kGy; (**c**) 300 kGy; (**d**) 600 kGy.

**Figure 23:**
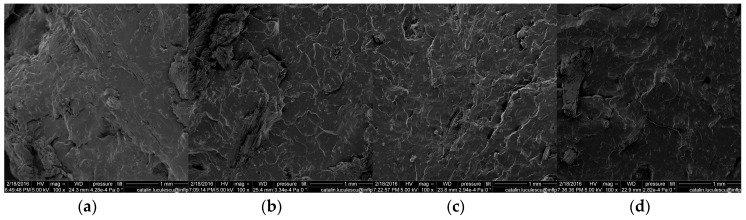
Scanning electron micrographs of the composites NR/20 phr wood sawdust, irradiated with (**a**) 75 kGy; (**b**) 150 kGy; (**c**) 300 kGy; (**d**) 600 kGy.

The surface of the specimen cryogenically fractured was investigated by SEM technique and the images are presented in Figure 21, Figure 22 and Figure 23. All the surfaces were placed on an aluminum mount, sputtered with gold palladium and then scanned at an accelerating voltage up to 30 kV.

Figure 21 shows that the fracture of the surfaces of the pure NR is characteristic for the brittle fracture on the cleavage plans, at all absorbed doses. Images presented in Figure 22 and Figure 23 and realized on the fracture surface of both composites based on NR and 10 and 20 phr wood sawdust show the absence of voids, which indicates a good interaction between the non-polar matrix and polar fillers. Moreover, because of the good adhesion between the matrix and filler, the blend deformation is not restricted by the presence of filler particle. Therefore, the absence of the voids and the aspect of the wood sawdust as being broken not snatched, in the samples analyzed by the SEM technique, coupled with the results of fiber-solvent interaction analysis, shows that the presence of the wood sawdust in the composites had improved the mechanical properties and cross-linking degree, due to the high compatibility of the wood sawdust with the natural rubber. The mechanical properties of the composites were modified, comparing with NR sample, by increasing the fiber concentration and irradiation dose. This means that the concentration of filler has not reached that point where poor adhesion between matrix and filler generates independent deformation of matrix leading to the restriction of the obtained material deformation [17,70].

## 4. Conclusions

Eco-composites, based on natural rubber and wood sawdust, were obtained by electron beam cross-linking. The electron beam irradiation doses were of 75, 150, 300 and 600 kGy and the concentration of wood sawdust in the composites was of 10 and 20 phr, respectively. The physical and mechanical properties of the obtained blends, except the elasticity and elongation set, were found as being superior to those of the pure natural rubber due to the addition of wood sawdust. Thus, the hardness, 100% Modulus, 300% Modulus, tearing and tensile strengths have increased with both irradiation dose and wood sawdust amount which indicate that the wood sawdust and rubber were in a good interaction and the wood sawdust has a reinforcing effect on the natural rubber. Elongation at break has decreased with the increase of EB dose and with the increase of wood sawdust amount in blends, indicating an increase in cross-link density. Elasticity (rebound resilience) has varied irregular whit the increasing of the wood sawdust amount and with the EB dose. The decrease in the resilience may be explained by the wood sawdust particles action that introduces a mechanism by which the strain energy diminishes. The gel fraction value, except for the natural rubber blend without wood sawdust and irradiated with 75 kGy, was over 90% for all prepared blends. The water uptake, swelling index and solubility have increased with increasing of fiber content. The cross-link density increases with the increasing of wood sawdust quantity in blends. All these results support our conclusion that the wood sawdust can be considered a good active fillers substitute for silica or carbon black that leads to the reinforcement of natural rubber.

## Figures and Tables

**Table 1:** *V_rf_* and *V_ro_*/*V_rf_* of NR/wood sawdust composites determined in toluene.

Amount of Wood Sawdust (phr)	*V_rf_*	*V_ro_*/*V_rf_*
	*75 kGy*	
10	0.0542	0.6602
20	0.0579	0.6182
	*150 kGy*	
10	0.0823	0.9924
20	0.0920	0.8875
	*300 kGy*	
10	0.1364	0.7164
20	0.1402	0.6968
	*600 kGy*	
10	0.1736	1.0388
20	0.1778	1.0141

**Table 2:** The compositional characteristics, designation and *p*_0_/*q*_0_ for NR/wood sawdust blends.

Wood Sawdust (phr)	*p*_0_/*q*_0_
0	0.2054
10	0.2305
20	0.1963
